# The Norwegian preeclampsia family cohort study: a new resource for investigating genetic aspects and heritability of preeclampsia and related phenotypes

**DOI:** 10.1186/s12884-015-0754-2

**Published:** 2015-12-01

**Authors:** Linda Tømmerdal Roten, Liv Cecilie Vestrheim Thomsen, Astrid Solberg Gundersen, Mona Høysæter Fenstad, Maria Lisa Odland, Kristin Melheim Strand, Per Solberg, Christian Tappert, Elisabeth Araya, Gunhild Bærheim, Ingvill Lyslo, Kjersti Tollaksen, Line Bjørge, Rigmor Austgulen

**Affiliations:** Department of Laboratory Medicine, Children’s and Women’s Health, the Norwegian University of Science and Technology (NTNU), 7491 Trondheim, Norway; Central Norway Regional Health Authority, 7501 Stjørdal, Norway; Department of Obstetrics and Gynecology, Haukeland University Hospital, 5058 Bergen, Norway; Department of Clinical Medicine, University of Bergen, 5020 Bergen, Norway; The Regional Biobank of Central Norway, St. Olavs Hospital, Trondheim, Norway; Department of Cancer Research and Molecular Medicine, NTNU, 7491 Trondheim, Norway; Department of Immunology and Transfusion Medicine, St. Olavs Hospital, 7006 Trondheim, Norway; Department of Obstetrics and Gynecology, Levanger Hospital, 7601 Levanger, Norway; Department of Obstetrics and Gynecology, St. Olavs Hospital, 7006 Trondheim, Norway; Department of Obstetrics and Gynecology, Stavanger University Hospital, 4068 Stavanger, Norway

**Keywords:** Preeclampsia, Family-based cohort, Genetic predisposition, Pregnancy

## Abstract

**Background:**

Preeclampsia is a major pregnancy complication without curative treatment available. A Norwegian Preeclampsia Family Cohort was established to provide a new resource for genetic and molecular studies aiming to improve the understanding of the complex pathophysiology of preeclampsia.

**Methods:**

Participants were recruited from five Norwegian hospitals after diagnoses of preeclampsia registered in the Medical birth registry of Norway were verified according to the study’s inclusion criteria. Detailed obstetric information and information on personal and family disease history focusing on cardiovascular health was collected. At attendance anthropometric measurements were registered and blood samples were drawn. The software package SPSS 19.0 for Windows was used to compute descriptive statistics such as mean and SD. P-values were computed based on *t*-test statistics for normally distributed variables. Nonparametrical methods (chi square) were used for categorical variables.

**Results:**

A cohort consisting of 496 participants (355 females and 141 males) representing 137 families with increased occurrence of preeclampsia has been established, and blood samples are available for 477 participants. Descriptive analyses showed that about 60 % of the index women’s pregnancies with birth data registered were preeclamptic according to modern diagnosis criteria. We also found that about 41 % of the index women experienced more than one preeclamptic pregnancy. In addition, the descriptive analyses confirmed that preeclamptic pregnancies are more often accompanied with delivery complications.

**Conclusion:**

The data and biological samples collected in this Norwegian Preeclampsia Family Cohort will provide an important basis for future research. Identification of preeclampsia susceptibility genes and new biomarkers may contribute to more efficient strategies to identify mothers “at risk” and contribute to development of novel preventative therapies.

## Background

Preeclampsia is a serious complication specific to human pregnancy affecting 3–5 % of all pregnant women in Western countries [1]. Although the condition has been documented already in ancient civilisations and has been subject to extensive research, preeclampsia is still a major cause of maternal and fetal morbidity and mortality worldwide. The course of the disease is unpredictable and it may rapidly progress to potentially life-threatening seizures and organ failure. There is no curative treatment, and delivery of the fetus and the placenta is the only way to alleviate severe symptoms, irrespective of gestational age. Aspirin is used as preventive medication in high-risk pregnancies. However, the reduced incidence of preeclampsia is only about 10 % [2]. In addition, the benefits for women at moderate risk of preeclampsia are not as clear. In order to develop effective preventive strategies and diagnostic tests and markers for preeclampsia, we need to increase our understanding of the complex pathophysiology of this disease.

The exact pathophysiological mechanisms of preeclampsia are not fully understood, however a model with two main stages including both placental and maternal factors has become widely accepted [3–5]. This conventional model proposes that abnormal development of the placenta results in reduced placental perfusion (stage 1) and release of placental factors into the maternal circulation leading to the clinical maternal manifestations of preeclampsia (stage 2). There is now a growing realization that preeclampsia may be classified as a syndrome and that there may be several different preeclampsia phenotypes (such as mild, severe, early onset, late onset, maternal, placental, recurrent). It has been suggested that different phenotypes may be distinguished by both clinical presentation and by distinct biomarkers. Defining different preeclampsia phenotypes by clinical and biochemical criteria in molecular studies may in the future lead to more specific therapeutic approaches [6].

Family-based studies have been the cornerstone of identification and quantification of familial risk and heritability of human diseases, aiming to evaluate whether familial clustering among cases is greater than expected. Epidemiological studies have shown familial clustering of preeclampsia implying a genetic component of the disease [7–14]. Family studies have underpinned the important role of maternal genes in the development of preeclampsia. Genome-wide linkage analyses have uncovered evidence for several maternal susceptibility loci in different populations [15–19]. Genetic factors are found to be responsible for approximately 50 % of the disease liability [18, 20]. By identification of genetic factors conferring susceptibility to preeclampsia one may contribute to elucidating the complex pathophysiology. Increased knowledge of the role played by genetic factors is also expected to increase our understanding of environmental contributions [21]. The establishment of the present cohort was inspired by the promising results from genome-wide scans in preeclampsia families from Iceland and Australia/New Zealand published in 1999 [15] and 2000 [18], respectively.

We aimed to establish a cohort of Norwegian families with an increased occurrence of preeclampsia in order to study the complex genetic architecture of this major pregnancy specific syndrome. The planning was independent of any specific genetic hypotheses. Our primary objective was to establish the cohort as a resource for future analysis.

## Methods

### Identification of participants

Recordings in the Medical birth registry of Norway (MBRN) from 1967 to 2005 was the basic data set used to identify the potential index women to be invited to the study. In Norway a notification form is sent to the MBRN for all births by the midwife/doctor. This notification includes information about biographical data of the child’s parents, maternal health conditions before and during pregnancy, complications during pregnancy or at birth and the newborn’s health condition. For the present study cohort we were interested in identifying women with a familial predisposition for preeclampsia. Familial predisposition was predefined as preeclamptic women with a first degree relative (mother, daughter or sister) also registered with preeclampsia in MBRN. Thus, a preeclampsia family in our cohort would have at least two affected women (potential index women). Mother-daughter and sister-sister index pairs were identified by linking MBRN data with The National Population Register. Furthermore, the list of potential index women was restricted to only include pairs of preeclamptic women who had delivered at the hospitals involved in the study.

**Fig. 1:**
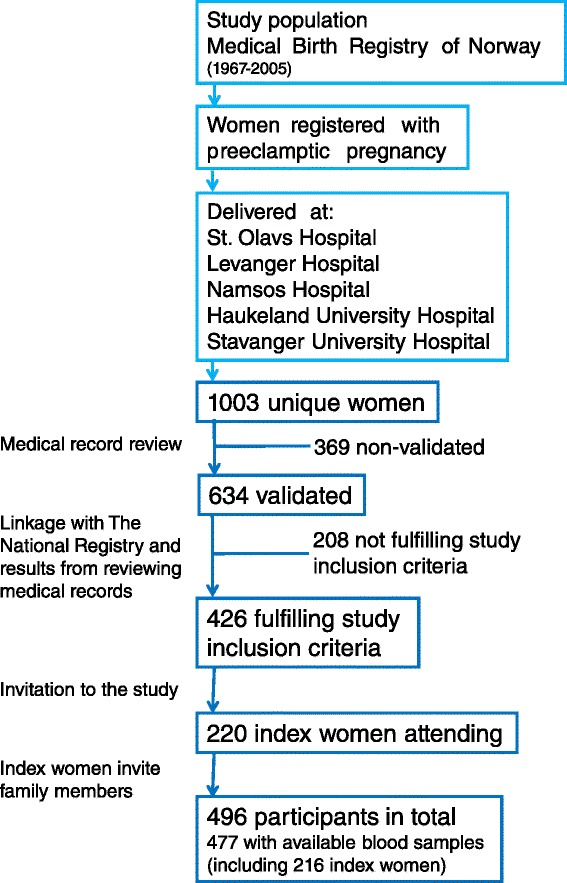
Flow chart summarizing the process of identification, validation of diagnoses and inclusion/participation

Medical records of the identified potential index women (*n* = 1003) were reviewed by medical doctors at the delivery wards involved in the study in order to assure recruitment of women with a valid preeclampsia diagnosis according to the diagnosis criteria set by The Norwegian Society of Gynecology and Obstetrics (NGF) (Table 1). Departments of Obstetrics and Gynaecology at the following hospitals were involved in the study: St. Olavs University Hospital, Haukeland University Hospital, Stavanger University Hospital, Levanger Hospital, Namsos Hospital (Fig. 1). Lists of women with confirmed valid diagnoses (i.e. index women) (*n* = 634) and non-valid diagnoses (*n* = 369) were returned from the departments of Obstetrics and Gynaecology to MBRN. At MBRN the lists were edited by exclusion of women without a valid preeclampsia diagnosis. Furthermore the sister/mother/daughter of these women was also excluded. Women being dead or where contact information was missing were removed from the lists. Thus, only alive mother-daughter or sister-sister pairs where both women had a valid preeclampsia diagnosis were invited to attend the study. The final number of index women fulfilling our inclusion criteria was 426 of 634 (67.2 %) [22]. Inclusion criteria are summarized in Table 1.

**Table 1:** Study inclusion criteria

Inclusion to:	Criteria				
	A: Blood pressure ≥140/90 > 20 weeks gestation	B: Proteinuria ≥0.3 g/L per 24 h or ≥ +1 on dipstick	C: Two measurements of hypertension and proteinuria	≥ first degree relative registered with preeclampsia in MBRN	≥ first degree relative with valid preeclampsia diagnosis
Examination of medical hospital record prior to invitation	x	x		x	
Invitation to attend the study	x	x	x	x	x

Criteria A, B and C together constitute the NGF diagnosis criteria for preeclampsia

### Recruitment of participants

The personal identification number, name, address and telephone number to all the 426 index women was forwarded from MBRN to the hospitals involved in the study. In order to motivate potential participants to attend the study we aimed to attract attention to our study by appearance in both national and local media (newspapers, radio and television) prior to posting invitation letters. From February 2009 to May 2011 the 426 index women were contacted from the involved delivery departments. As paternal genetic factors appear to contribute to preeclampsia susceptibility [23, 24] the invited index women were encouraged to recruit their male partner(s) fathering their pregnancies. Index women agreeing to participate were also encouraged to recruit other female and male family members. Index women who had delivered at St. Olavs Hospital in Trondheim were the first to be recruited to the study. The postal invitations from St. Olavs Hospital included the invitation letter, informed consent form, information to family members, contact information and a questionnaire. Due to the responses we got from the women recruited from St. Olavs Hospital the procedure of posting invitations from the other involved delivery wards were organized differently. A post card notifying women that they were going to be invited to the study and information regarding local media appearance were sent first. One week later the second postal item containing the invitation letter, informed consent form, information to family members, contact information and information about what to do if they agreed to attend was sent. Women giving their consent subsequently received the questionnaire. Non-responders were either sent a postal reminder or were contacted by phone.

A flow chart summarizing the process of identification, validation of diagnoses and inclusion/participation is shown in Fig. 1.

### Data collection

All participants were invited to fill in a questionnaire, get their height, weight and waist circumference measured and donate blood samples. The questionnaire was accomplished in collaboration with a medical doctor. In addition, obstetric information was collected from medical records for index women who received postal invitation.

#### Questionnaire

A questionnaire was designed by researchers and medical doctors in the project team establishing this Norwegian Preeclampsia Family Cohort Study. The questionnaire covers the following main topics: biographic information, relevant information regarding pregnancies, self-reported personal somatic health and diseases such as diabetes, coronary heart disease, stroke and asthma, family and family in-law health/disease history. Detailed obstetric information was collected from all female participants. A summary of the type of data collected is shown in Table 2.

**Table 2:** Overview of data collected from participants

1. Questionnaire	
a.Maternal characteristics	Age at attendance, exposed for preeclampsia, parity, smoking habits and medication during pregnancy
b.Paternal characteristics	Age at attendance, exposed for preeclampsia, fathered a preeclamptic pregnancy
c.Pregnancy and birth characteristics	Due date, date of birth, preeclampsia, gestational hypertension, gestational diabetes, eclampsia, HELLP, mode of delivery (vaginal or cesarean section), labor onset (spontaneous or induction), placental weight, experienced abortions
d.Offspring characteristics	Sex, birth weight, twin or multiple
e.Self-reported personal disease history focusing on preeclampsia, diabetes and cardiovascular disease	Time of onset of disease, duration of disease
f.Self-reported family disease history focusing on preeclampsia, diabetes and cardiovascular disease	
2. Physical measurements at attendance	
a.Height	
b.Weight	
c.Waist circumference	
3. Biological samples (blood)	
a.EDTA – 1 × 10 ml	Buffy coat for DNA analyses (genetic and epigenetic), plasma for protein/metabolites/nutrients analyses
b.Serum Separating Tube (SST) – 1 × 10 ml	Serum for protein/metabolites/nutrients analyses
c.Tempus Blood RNA tubes – 1 × 9 ml (6 ml RNA stabilizing fluid)	Whole blood for RNA analyses (gene expression and qualitative analyses)

#### Biospecimens

Blood samples for analysis at different levels in the biological process of converting DNA to intermediate RNA and executive proteins were collected. Three different peripheral blood samples were collected from all participants; 1) EDTA whole blood sample (Puls-Norge), 2) Tempus™ Blood RNA Tube (Applied Biosystems) sample and 3) Serum Separating Tube (SST) (Puls-Norge) sample (Table 2). Buffy coat and plasma were extracted from the EDTA blood sample and divided into aliquots. Extraction of DNA from the buffy coats is accomplished, and DNA is available for genetic studies. Serum was extracted from the SST samples and divided into aliquots, whereas the Tempus™ Blood RNA Tubes containing a RNA-stabilization reagent are stored until extraction of RNA. All aliquots have been co-localized and are stored at HUNT biobank (http://www.ntnu.edu/hunt/hunt-biobank) in Levanger at −80 °C.

### Linkage to routine data sources

The current cohort has a potential for follow-up studies. However, only passive follow-up studies by linkage of data from The Preeclampsia Family Cohort Study with data from local, regional and national end-point registers and routine data sources has been planned. Relevant registers for follow-up studies in the presented cohort are MBRN, Cause of Death Register and local/regional hospital disease registers e.g. on myocardial infarction and stroke. Linkage to health registers is possible through the unique Norwegian 11-digit personal identification number after approval from the Regional Committee for Medical and Health Research Ethics.

### Statistical analyses

We compared maternal age at birth, placenta weight, birth weight in preeclamptic pregnancies versus non-preeclamptic pregnancies for the index women by using independent sample *t*-test (two tailed) (significance level 0.01). Pearson chi square analysis in a 2 × 2 contingency table was performed when comparing number of cesarean sections, number of induced vaginal pregnancies with the number of non-induced vaginal deliveries, the number of acute with the number of planned cesarean sections and the number of male with the number of female neonates in preeclamptic pregnancies versus non-preeclamptic pregnancies among the index women (significance level 0.05). All statistical analyses were performed in IBM SPSS Statistics 19.

#### Power in genetic studies based on The Preeclampsia Family Cohort Study

**Fig. 2:**
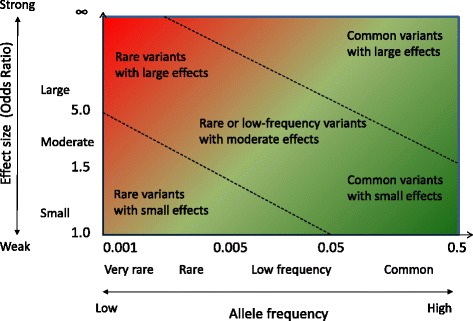
Relationship between effect size and allele frequency (adopted from [25, 26]). Extremely rare genetic variants with large effect sizes (*upper left, strong red color*) are often identified in family-based genome-wide linkage analyses. Common genetic variants with small effect sizes (*lower right, strong green color*) have been identified in traditional GWAS (including only common variants). Rare variants with small effects (*lower left*) are difficult to identify. Whereas common genetic variants with large effects (*upper right*) have been identified using both linkage analysis and GWAS, however these are highly unusual for common diseases

**Fig. 3:**
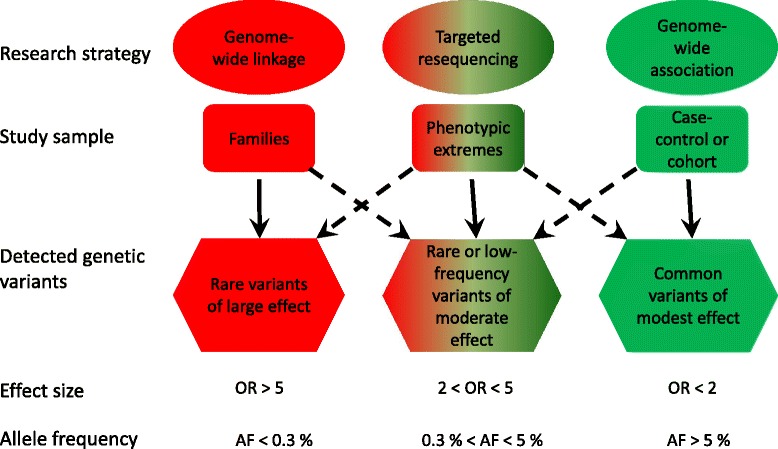
Primary research strategies for identification of genetic variants across the allele frequency spectrum (adopted from [27]). Genome-wide linkage studies are well suited to identification of genetic variants with allele frequencies below 0.3 % with large effect sizes (OR > 5). Targeted resequencing often leads to identification of genetic variants with allele frequencies between 0.3 and 5 % with moderate effect sizes (2 < OR < 5), but may also be used to identify rare variants with large effects and common variants with modest effects. Traditional GWAS is suited to identification of common genetic variants with modest effect sizes (OR < 2)

In general, the power to detect the effect of genes depends on the effect size, the allele frequency and the sample size. The ability to detect the genes (genetic susceptibility variants) increases when sample size and effect size increase. However, the choice of study design and research strategy is still crucial for the chances of a successful outcome in a genetic study. Figure 2 (adopted from [25, 26]) shows the relationship between effect size and allele frequency. Figure 3 (adopted from [27]) shows what genetic variants that may be detected with three primary strategies (genome-wide linkage, targeted sequencing and genome-wide association). Genome-wide linkage analyses can only be performed in cohorts/collections of biologically related individuals and are preferred when searching for rare variants (allele frequency <0.3 %) expected to have large effect size (odds ratio (OR) > 5). Whereas, genome-wide association studies (GWAS) in case-control or cohort studies, are better suited to identify common variants (allele frequency >5 %) of modest effect size (OR < 2).

The presented cohort is family-based and well suited for genome-wide linkage analysis. Other research strategies such as targeted resequencing and family-based association analyses are also possible. The present cohort consists of several nuclear families, and genetic studies of these are likely to represent a more homogeneous and limited set of causative genes and pathways. In addition, we have performed thorough phenotyping of the cohort [28]. These features are likely to enhance statistical power for gene discovery in our cohort. Furthermore, significant heritability estimates in the cohort support that there is an increased susceptibility of preeclampsia [28]. High heritability implying a strong correlation between phenotype and genotype makes it easier to detect loci with an effect on the trait. However, further research is needed as heritability does not provide information about the genetic architecture.

### Ethical approval

The establishment of the Norwegian Preeclampsia Family Cohort Study was approved by the Regional Committee for Medical Research Ethics Mid-Norway, the National Data Inspectorate and The Directorate of Health and Social Welfare in Norway. All participants gave their informed consent when attending the study.

### Data access

The Preeclampsia Family Cohort Study data and biological material are held by the research team at Haukeland University Hospital, Bergen Health Authority and the Department of Cancer Research and Molecular Medicine, Faculty of Medicine, Norwegian University of Science and Technology (NTNU). Access to the data is not freely available. However, The Preeclampsia Family Cohort Study data and biological material have the potential to be subject of many internally and externally collaborations. Potential collaborators should discuss ideas informally with the person in charge of the cohort study, Line Bjørge (Line.Bjorge@uib.no). Specific proposals for collaborative ideas are welcome and when relevant approvals are consented research projects should be conducted in collaboration with appropriate members of The Preeclampsia Family Cohort Study team. The Preeclampsia Family Cohort Study is completely researcher-driven and funded from several research grants, thus all potential collaborators must cover all costs related to potential analyses.

## Results

### Sample size and response fractions

A participant is a person who has given a written informed consent, donated blood samples and/or been interviewed and who’s questionnaire has been filled in. The total participation rate for all eligible index women was 51.6 % (220/426). Of the eligible mother-daughter pairs 42.2 % (18/42) attended the study, whereas 38.8 % (64/165) of the eligible sister-sister pairs attended (Table 3). In total there are 496 participants in the Preeclampsia Family Cohort Study, 355 female and 141 male participants, whereas blood samples are available for 477 participants. Of the participating index women 37.3 % (82/220) recruited their husbands/partners to attend the study. Other male relatives also attended, and 15 of them reported that they had fathered a preeclamptic pregnancy. Of the male participants fathering preeclamptic pregnancies 23 also have an offspring participating in the study, enabling analyses of relationships between mother, father and child.

**Table 3:** Number of eligible and participating index women and families in the Preeclampsia Family Cohort Study

	Invited	Attending	Participation rate (%)
Index women	426	220	51.6
Mother-daughter pairs	42	18	42.9
Sister-sister pairs	165	64	38.8
Three sisters	5	1	20.0
Familiy with at least two index women participating	209	81	38.8
Familiy represented in the cohort by one or more participants^a^	209	137	65.6

^a^In 56 families only one of the eligible index women participated

### Characteristics of participants

Preeclampsia is a heterogeneous condition and defining different preeclampsia phenotypes by clinical criteria is likely to improve the chances of finding molecular factors distinguishing these phenotypes. A comprehensive characterization of phenotypic subgroups in the total cohort has been carried out and is presented in a separate publication recently published. In this publication heritability of different preeclampsia phenotypes and also related conditions such as atherothrombotic cardiovascular diseases (aCVD) has been estimated in the present cohort [28].

#### Descriptive statistics of index women

Descriptive statistics of index women and their pregnancies are presented in Table 4. About 60 % (326/547) of the index women’s pregnancies with birth data registered was preeclamptic according to NGF diagnosis criteria (Table 4). Among the index women 40.9 % (90/220) experienced more than one preeclamptic pregnancy (recurrent preeclampsia). It is generally known that preeclamptic pregnancies are more often accompanied with delivery complications. As expected the number of cesarean sections compared with the number of vaginal deliveries was found to be significantly higher in preeclamptic pregnancies versus non-preeclamptic pregnancies (*p* = 3.2 × 10^−5^) in our cohort (Table 4). The number of induced vaginal deliveries was also found to be significantly higher in preeclamptic pregnancies compared with the non-preeclamptic pregnancies (*p* = 3.2 × 10^−27^) (Table 4). Furthermore, acute cesarean sections are significantly more frequent than planned cesarean sections in preeclamptic pregnancies compared with non-preeclamtic pregnancies (*p* = 5.6 × 10^−4^). A significance level of 0.05 was set when performing the Pearson chi square tests. Placenta weight and birth weight was found to be significantly lower in preeclamptic pregnancies compared with non-preeclamptic pregnancies (*p* = 0.9 × 10^−5^ and *p* = 1.9 × 10^−15^ respectively) as expected (Table 4) at a significance level of 0.01.

**Table 4:** Selected clinical characteristics of index women and their pregnancies

	Pregnancies among index women	Preeclamptic pregnancies among index women	Non-preeclamptic pregnancies among index women
	(Pregnancies with birth data *n* = 547) (Live birth pregnancies *n* = 544)	(*n* = 326)	(*n* = 214)
Age at delivery no. (mean ± standard deviation)			
1	24.5 ± 4.9 (*n* = 214)	24.8 ± 5 (*n* = 181)	23.1 ± 4.0 (*n* = 32)
2	27.7 ± 4.5 (*n* = 194)	28.2 ± 4.4 (*n* = 89)	27.3 ± 4.7 (*n* = 103)
3	30.6 ± 4.6 (*n* = 95)	31.1 ± 4.9 (*n* = 44)	30.2 ± 4.4 (*n* = 51)
4	31.9 ± 3.2 (*n* = 27)	31.5 ± 4.1 (*n* = 10)	32.1 ± 2.6 (*n* = 17)
5	33.2 ± 4.2 (*n* = 9)	31.5 ± 6.4 (*n* = 2)	33.7 ± 3.9 (*n* = 7)
Multiple pregnancy	*n* = 7	*n* = 4	*n* = 3
Mode of delivery			
Vaginal^a,b^	*n* = 396	*n* = 215	*n* = 178
Induced^b^	*n* = 196	*n* = 160	*n* = 35
Cesarean section^a^	*n* = 136	*n* = 102	*n* = 34
Planned^c^	*n* = 37	*n* = 20	*n* = 17
Acute^c^	*n* = 99	*n* = 82	*n* = 17
Placenta weight^d^	591 ± 164 (264 valid, 283 missing)	560 ± 166 (173 valid, 155 missing)	653 ± 138 (89 valid, 125 missing)
Birth weight^e^	3189 ± 843 (528 valid, 19 missing)	2981 ± 874 (321 valid, 7 missing)	3526 ± 645 (204 valid, 10 missing)
Neonate sex			
Male	*n* = 261	*n* = 152	*n* = 106
Female	*n* = 288	*n* = 180	*n* = 108
Experienced abortions			
Spontaneous	*n* = 89		
Induced	*n* = 43		

^a^Comparing the number of vaginal deliveries with the number of cesarean sections in preeclamptic pregnancies versus non-preeclamtic pregnancies there is a significant difference using Pearson’s chi square analysis in a 2 × 2 contingency table. The number of cesarean sections was significantly higher in preeclamptic pregnancies (*p* = 3.2 × 10^−5^)

^b^Comparing the number of induced vaginal deliveries with the number of non-induced vagnial deliveries in preeclamptic pregnancies versus non-preeclamtic pregnancies there is a significant difference using Pearson’s chi square analysis in a 2 × 2 contingency table. The number of induced vaginal deliveries was significantly higher in preeclamptic pregnancies (*p* = 3.2 × 10^−27^)

^c^Comparing the number of acute cesarean sections with the number of planned cesarean sections in preeclamptic pregnancies versus non-preeclamtic pregnancies there is a significant difference using Pearson’s chi square analysis in a 2 × 2 contingency table. The number of acute cesarean sections was significantly higher in preeclamptic pregnancies (*p* = 5.6 × 10^−4^)

^d^The placenta weight in preeclamptic pregnancies was significantly lower compared with non-preeclamptic pregnancies using *t*-test statistics (*p* = 0.9 × 10^−5^)

^e^The birth weight of neonates born from preeclamptic pregnancies was significantly lower compared with non-preeclamptic pregnancies using *t*-test statistics (*p* = 1.9 × 10^−15^)

#### Distribution of non-gestational diseases related to preeclampsia in index women

As shown in Table 2, self-reported information on non-gestational disease phenotypes related to development of preeclampsia was collected. Definition of and proportion of disease phenotypes in the total cohort is presented in a paper by Thomsen et al. [28]. Whereas, the proportion of diabetes, pulmonary disease, autoimmune (including systemic lupus erythematosus), aCVD, kidney disease, chronic hypertension, hypercholesterolemia, myocardial infarction/angina, stroke and thrombosis in the index women is shown in Table 5. As in the total cohort, aCVD (30.7 %) and chronic hypertension (25.1 %) were the most prevalent disease phenotypes in index women.

**Table 5:** Distribution of non-gestational diseases related to development of preeclampsia in index women

Disease phenotype	Proportion (%)
Diabetes mellitus type 1	1/214 (0.5)
Diabetes mellitus type 2	9/213 (4)
Gestational diabetes	15/214 (7.0)
Kidney disease	3/215 (1.4)
Pulmonary disease	29/215 (13.5)
Autoimmune disease	26/214 (12.1)
aCVD	66/215 (30.7)
Chronic hypertension	54/215 (25.1)
Hypercholesterolemia	20/215 (9.3)
Myocardial infarction/Angina	3/215 (1.4)
Stroke	0/214 (0)
Thrombosis	7/215 (3.3)

*aCVD* atherothrombotic cardiovascular disease

## Discussion

We have successfully established a unique family-based cohort consisting of Norwegian families with increased occurrence of preeclampsia. All families were identified with at least two first degree related women with a valid preeclampsia diagnosis according to modern diagnosis criteria. One of the main strengths of the Preeclampsia Family Cohort Study is that it holds both biological material and detailed health-related information, and also the ability to collect complemental information from local, regional or national routine data sources. Thus, the established cohort represents a new resource for researching genetic and molecular aspects of preeclampsia, and also provides an important basis for translational research.

The descriptive statistics of the index women’s pregnancies presented in this paper did not show any unexpected results. We found that women experiencing preeclamptic pregnancies are significantly more affected by labor and delivery complications. Another observation was that many of the index women (40.9 %) have experienced more than one preeclamptic pregnancy. The most likely explanation for lower placenta and birth weight in preeclamptic compared with non-preeclamptic pregnancies is that these pregnancies usually are shorter.

It has been suggested that rare genetic variants may have a greater impact on disease development. Rare genetic variants with large effects on specific traits are likely to better help us understand the underlying biology of health and disease than common variants conferring moderate effect on complex traits [29–31]. Thus, attention to rare genetic variants has increased and led to a renaissance for family-based studies in the post-GWAS era since these variants are easier to identify with family-based designs. In addition, new high throughput sequencing technology has revolutionized the field of molecular genetic research by raising new opportunities to dissect common complex diseases such as preeclampsia.

Family-based cohorts are difficult and challenging to establish. In the presented cohort the total participation rate for all eligible index women was 51.6 % (220/426). The low participation rate is a limitation of the study as sample size affects the power to detect the effect of genes. Low participation rate may also lead to selection bias. However, in general family-based designs have several advantages compared to population-based designs in genetic studies [32]. Data on sib pairs, nuclear families and extended pedigrees can be used to make inferences in all of the major areas of genetics i.e. estimation of heritability of a trait, localize quantitative trait loci (QTLs) via linkage information and use association information to fine-map and identify QTLs (see Table 6) [32].

**Table 6:** Major types of sampling designs in human genetics and the types of genetic inferences that can be made (modified from [32])

Sampling design	Possible inferences		
	Heritability	Linkage	Association
Unrelated individuals	–	–	+
Triads (parents, one offspring)	–	+	+
Sibling pairs	+	+	+
Nuclear families	**+**	**+**	**+**
Extended pedigrees	+	+	+

An advantage of family-based designs is that they are robust against population substructure [33]. Significant findings always imply both linkage and association [34], and one therefore avoids false positives (Type I errors) that often arise when association is present but linkage is not. Family-based designs also have substantial benefits in terms of accounting for multiple-hypothesis testing, especially when hundreds of thousands of genetic markers are tested simultaneously, because they contain both within- and between-family information [35]. In addition, family data and material that have already been collected for genome-wide linkage studies may be recycled for association analyses, also in population-based studies [36, 37].

In family-based designs one has the ability to make use of familial correlations to increase power using strategies such as the “extreme discordant and concordant” design, obtain information on genetic haplotypes thus avoiding haplotype estimation/prediction and provide parent of origin information. This means that studies of families may have superior power to detect clinically relevant genetic variants compared to population-based cohort studies. Family-based designs focusing on identifying gene loci associated with important quantitative traits are also likely to complement population-based cohort studies and other studies with the ability to focus on gene-environment interactions. It has been suggested that family samples can provide substantially greater power for rare variant association studies because there is a potential for observing many copies of a rare genetic variant associated with a disease or trait. This has been called the “jackpot” effect [37].

In the present cohort study we have the ability to carry out the transmission-disequilibrium test (TDT), which is a simultaneous test for linkage and association. The classical TDT is a completely non-parametric test comparing transmitted with non-transmitted parental alleles. A major advantage of this test is the robustness to potential misspecification of any of the features of the disease model or trait/phenotype distribution since the validity of the test does not require proper specification of the mode of inheritance (disease model) or assumptions about the distribution of the disease in the population.

Preeclampsia is a complex and heterogeneous syndrome that is not only a maternal disease influenced by maternal genetic risk factors. Due to the placental origin of preeclampsia it is likely that fetal and/or paternal genetic factors play a central role in the pathogenesis. Evidence is supporting that paternal and/or fetal genetic factors might contribute to the risk of preeclampsia [23, 24, 38]. It has been shown that men born from preeclamptic pregnancies are at a higher risk of fathering a preeclamptic pregnancy [23], and men who have fathered a preeclamptic pregnancy have an increased risk of fathering another preeclamptic pregnancy with a different partner [24]. Thus, it is important to include the father and the offspring in genetic studies of preeclampsia. This may be possible in studies based on the Preeclampsia Family Cohort Study.

Family-based study designs allow for sub-designs, such as case-parent triads, that open the possibility of detecting maternal, paternal and fetal genes and also their interactions. Gene-environment interactions may also be detected in case-parent triads if stratified by environmental exposure. The majority of genetic studies of preeclampsia have focused on the maternal genetic contribution. This is also the case for genome-wide family linkage analyses. However, recently it has been shown that fetal susceptibility loci also may be detected by linkage analysis [39]. The repeated pregnancy design where the maternal genome is unchanged and the fetal genotype changes is another sub-design to examine maternal and fetal genotype and interaction with environmental exposures in family-based studies.

The fact that all families in the Norwegian Preeclampsia Family Cohort Study are identified with two “true” preeclamptic women, implying that there is a clear familial aggregation of the disease, strengthens the potential to uncover genetic factors contributing to development of preeclampsia. Significant heritability estimates in the cohort also support that there is an increased susceptibility of preeclampsia [28]. Relatively strict inclusion criteria were used in the identification process for our cohort in order to reduce phenotypic heterogeneity and improve power. However, as a result sample size was reduced at the cost of some statistical power.

Another weakness of cohorts with disease-based ascertainment is that studies based on these cohorts are essentially limited to the investigation of a single or only a small spectrum of phenotypes. Although the main phenotype in the present cohort is preeclampsia findings based on our cohort are likely to be relevant to other major health issues among women, such as cardiovascular disease (CVD). The reason for this is the association between preeclampsia and development of maternal CVD later in life which has been well established through epidemiological research [40–46]. Pathological features such as endothelial dysfunction and inflammation and also many common risk factors are shared between these conditions [44, 45, 47–51]. Furthermore, recent empirical evidence supporting the hypothesis that genetic risk factors underlying preeclampsia are shared with CVD-related traits is now emerging [52–55]. A history of preeclampsia has also been associated with subsequent maternal health risks including adverse effects on diabetes and cancers (reviewed in [40, 56]). In this regard, the present cohort study holds a unique feature namely the ability to link individual data with relevant and detailed morbidity, mortality and other routine data sources. Thus, follow-up studies may be carried out through linkage of the presented cohort with local, regional or national routine data sources and potentially also through review of medical hospital records.

A major weakness of cohorts including more than one generation is that blood samples most often are drawn independent of the onset of the disease/condition under study. As in the presented cohort the blood samples were not drawn when the women were pregnant. Thus, the utility of blood samples in family-based cohorts to study complex genetic diseases are somewhat limited to studies of genetic predisposition and less valuable for functional studies. However, studies in family-based cohorts may generate novel hypotheses that can be tested functionally in different human samples or in different model systems.

## Conclusions

The Norwegian Preeclampsia Family Cohort Study has recently been completed and made available for research. On-going genetic studies in the cohort remain to discover what genomic regions are linked to preeclampsia in the Norwegian families, and if these comply with previous linkage studies in Icelandic, Australian/New Zealand, Finnish and Dutch families. Furthermore, we aim to identify novel susceptibility genes, pathways and causal genetic variants in order to help understanding the pathophysiology of preeclampsia and also cardiovascular disease in women. The presented cohort provides an important basis for future genetic, molecular and epidemiological studies of preeclampsia and associated traits.
